# Effectiveness of antiviral treatment in HBeAg-negative chronic hepatitis B patients with normal or mildly elevated alanine aminotransferase: a retrospective study

**DOI:** 10.1186/s12876-022-02471-y

**Published:** 2022-08-17

**Authors:** Sufang Wei, Meixin Hu, Hongjie Chen, Qiuli Xie, Peng Wang, Hong Li, Jie Peng

**Affiliations:** 1 State Key Laboratory of Organ Failure Research, Guangdong Provincial Key Laboratory of Viral Hepatitis Research, Department of Infectious Diseases, Nanfang Hospital, Southern Medical University, Guangzhou, 510080 China; 2 Department of Infectious Diseases, Shunde Hospital, Southern Medical University, Foshan, 528300 China

**Keywords:** Chronic hepatitis B, Hepatitis B virus DNA, Alanine aminotransferase, Nucleoside/nucleotide analogues, FibroScan

## Abstract

**Background:**

There are inadequate data and no histological evidence regarding the effects of antiviral treatment for hepatitis B e-antigen (HBeAg)-negative chronic hepatitis B (CHB) patients with normal or mildly elevated alanine aminotransferase (ALT). This study investigated the effects of antiviral treatment on these patients.

**Methods:**

We retrospectively analysed the outcomes of antiviral treatment for HBeAg-negative CHB patients with normal or mildly elevated ALT who were treated with nucleoside/nucleotide analogues (NAs) for up to 96 weeks.

**Results:**

A total of 128 patients were enrolled; 74 patients had normal ALT and 54 patients had mildly elevated ALT. The total cumulative rates of viral suppression were 64.06%, 81.97%, and 96.39%, at weeks 24, 48, and 96, respectively. The cumulative rates of viral suppression for the normal and mildly elevated ALT groups were 67.85% and 58.97%, 86.39% and 76.31%, and 93.13% and 97.04% at weeks 24, 48, and 96, respectively. The serum HBV DNA levels at week 12 and hepatitis B surface antigen (HBsAg) levels at week 24 were significant predictors of the 96-week virological response. Of the 128 patients, 54 with normal ALT and 33 with mildly elevated ALT underwent FibroScan at baseline. Significant fibrosis (F ≥ 2) was found in 44.4% (n = 24) and 51.5% (n = 17) of the patients in the normal ALT group and mildly elevated ALT group, respectively. Compared with the values at baseline, liver stiffness values significantly decreased at week 48 (8.12 kPa vs. 6.57 kPa; *p* < 0.001) and week 96 (8.87 kPa vs. 6.43 kPa; *p* < 0.001), respectively.

**Conclusions:**

HBeAg-negative CHB patients with normal ALT could benefit from antiviral therapy with NAs, similar to patients with mildly elevated ALT. Antiviral treatment is strongly recommended for HBeAg-negative CHB patients with normal ALT. Additionally, significant liver fibrosis is not rare in HBeAg-negative CHB patients with ALT less than two-times the upper limit of normal, and FibroScan should be performed regularly for these patients.

**Supplementary Information:**

The online version contains supplementary material available at 10.1186/s12876-022-02471-y.

## Background

Chronic hepatitis B virus (HBV) infection remains a major public health problem worldwide and can lead to cirrhosis and hepatocellular carcinoma (HCC), which contribute to high mortality and morbidity among humans [1]. The natural history of chronic HBV infection is variable, but it is generally divided into the following five stages based on the hepatitis B e-antigen (HBeAg) status, serum HBV DNA levels, alanine aminotransferase (ALT) levels, and liver disease severity stage: HBeAg-positive chronic HBV infection; HBeAg-positive chronic hepatitis B (CHB); HBeAg-negative chronic HBV infection; HBeAg-negative CHB; and hepatitis B surface antigen (HBsAg)-negative phase [2]. HBeAg-negative CHB is caused by replicative HBV mutants that fail to produce HBeAg (precore mutants) or downregulated precore/core messenger RNA transcripts (basic core promoter mutants) [3, 4]. HBeAg-negative CHB develops during the course of HBeAg-positive chronic HBV infection or after the loss of HBeAg and its seroconversion to the anti-HBe stage; this is becoming the predominant form of CHB in many countries worldwide. One study revealed that precore mutants in HBeAg-negative CHB patients increased the rate of recurrence after initially effective interferon therapy [5]. Another study revealed a negative correlation between the number of mutations in the promoter region of the basic C gene and the effectiveness of interferon therapy for HBeAg-negative CHB [6]. Additionally, basic core promoter mutations and precore mutations directly impact the replication capacity of Lamivudine-resistant mutants [7]. Increasing data support that HBeAg-negative CHB patients with precore mutants and/or basic core promoter mutants are at higher risk for serious liver disease and HCC than HBeAg-positive CHB patients [8–10].

Ideally, an adult patient with chronic HBV infection should be treated early to improve the chance of survival by preventing disease progression. Nevertheless, to date, determining treatment initiation for patients with chronic HBV infection has been based on serum ALT levels. Antiviral therapy is recommended for HBeAg-negative CHB patients with ALT two-times or more than the upper limit of normal (ULN) and ALT at or more than the ULN with or without significant necroinflammation or fibrosis; however, according to the current guidelines, regular monitoring is suggested for HBeAg-negative CHB patients with ALT less than the ULN [2, 11, 12]. Antiviral treatment is not indicated for HBeAg-negative CHB patients with normal ALT for having the following: a low incidence of histological progression, high rates of HBsAg clearance, and very low risks of cirrhosis and HCC [13]. Although ALT elevation remains a critical indicator of hepatic necroinflammation [14], an increasing number of studies revealed that significant abnormalities of the liver tissues frequently occur in patients without significantly elevated ALT [15, 16]. Previous trials detected necroinflammation in 11.8% of patients with normal ALT and 22.2% of patients with mildly elevated ALT, whereas their incidence rates of serious fibrosis were 49.0% and 55.6%, respectively [17]. The reported incidence of significant liver disease for HBeAg-negative CHB patients is 38.2% [18]. Moreover, some studies have found that untreated HBeAg-negative CHB patients with normal or mildly elevated ALT are at an increased risk for HCC and death [19, 20].

Nucleoside/nucleotide analogues (NAs) are first-line therapeutic drugs for the treatment of CHB patients according to most clinical practice guidelines, and they have been used because of their robust antiviral activity and safety [21, 22]. There is evidence that NAs can prevent the transmission of HBV as well as infections of transplanted livers by reducing viral loads and producing non-infectious variations of HBV virions [23].

It is crucial to accurately estimate the severity of liver fibrosis in CHB patients because liver fibrosis is an essential prognostic factor for liver disease. Liver biopsy is regarded as the gold standard for assessing fibrosis or cirrhosis in CHB patients; however, its clinical application is often constrained because of its invasiveness and cost-effectiveness. Therefore, non-invasive substitutions are required to assess the degree of liver fibrosis. The use of FibroScan for performing liver stiffness measurements (LSMs) has been recommended as a reliable and non-invasive technique for evaluating liver fibrosis [11, 24, 25]. The accuracy of FibroScan for diagnosing different liver fibrosis stages has been shown to be excellent [26]. When used to distinguish significant fibrosis (F ≥2), FibroScan had an area under the receiver-operating characteristic curve, sensitivity, and specificity of 0.87, 81%, and 82%, respectively. When distinguishing cirrhosis (F=4), FibroScan had an area under the receiver-operating characteristic curve, sensitivity, and specificity of 0.89, 93%, and 82%, respectively. A recent report by Kim et al. [27] demonstrated that FibroScan can recognize CHB patients at high risk for HCC without clinical evidence of liver cirrhosis. These results illustrated the importance of FibroScan for assisting clinicians with the management of CHB patients. Therefore, in the present study, we used FibroScan to estimate the histological improvement of these patients.

To date, however, there are insufficient data and no histological evidence regarding the antiviral outcomes of NAs for HBeAg-negative CHB patients with normal or mildly elevated ALT. We evaluated the efficacy of antiviral therapy for patients with ALT less than two-times the ULN in China.

## Methods

### Patients

A total of 128 HBeAg-negative CHB patients from Nanfang Hospital and Shunde Hospital of Southern Medical University in Guangdong Province, China, between 2010 and 2020, were enrolled in this retrospective study. The selection criteria were as follows: HBV DNA load ≥ 2000 IU/ml; serum HBsAg positivity for more than 6 months; patients who were HBeAg-negative and had received monotherapy with entecavir, tenofovir disoproxil fumarate, and tenofovir alafenamide; ALT levels less than two-times the ULN within 6 months consecutively (a cutoff of 40 U/L was used for serum ALT levels); and treatment with NAs only without interruption. The exclusion criteria were as follows: co-infection with hepatitis C virus or human immunodeficiency virus; decompensated cirrhosis; HCC at enrollment or a history of HCC; other chronic liver diseases; cardiovascular disease, cancer, autoimmune disorders or renal dysfunction; pregnancy; and alcoholism.

The CHB patients were divided into the normal ALT group (n = 74) and mildly elevated ALT group (n = 54) according to the baseline serum ALT levels. The study design was approved by the appropriate ethics review board of Nanfang Hospital and Shunde Hospital. Written informed consent to use the clinical data for this study was obtained from all patients; their information was anonymised and de-identified prior to analysis. All methods were performed in accordance with the relevant guidelines and regulations.

### Virologic and liver function tests

We reviewed the medical records and collected the laboratory data of all patients. First, the serum HBV DNA load was measured using a real-time polymerase chain reaction. Second, HBeAg and anti-HBe levels were determined using commercially available enzyme immunoassays (Alisei Quality System; RADIM, Rome, Italy). Third, serum HBsAg levels were quantitatively measured using Elecsys HBsAg II immunoassays (Roche Professional Diagnostics, Rotkreuz, Switzerland). Fourth, liver biochemistry, including serum ALT, AST, albumin, total bilirubin, and direct bilirubin levels were examined using the colorimetric method (MODULAR EVO; Hoffmann-La Roche Ltd., Basel, Switzerland). Finally, parameters of the haematopoietic function, including the white blood cell count, platelet count, lymphocyte count, and hemoglobin count, were recorded.

### Liver stiffness measurements using FibroScan

A FibroScan device (Echosens, Paris, France) was used by trained operators according to the manufacturer’s instructions to assess the severity of liver fibrosis for each patient. Paired LSMs were obtained before and after antiviral therapy. The median value of at least 10 successful measurements was considered reliable. The results are expressed as kilopascals (kPa). Liver stiffness values ≥ 7.3 kPa indicated significant fibrosis.

### Study endpoints

The primary efficacy endpoint of this study was the cumulative incidence of the virological response (VR), which was defined as undetectable HBV DNA in patients with chronic HBV infection. The secondary outcomes were decreased HBV DNA and HBsAg levels, loss rate of HBsAg, biochemical response, and decreased LSMs. We defined HBsAg loss as HBsAg < 0.05 IU/ml.

### Statistical analysis

Statistical analysis was performed using the Statistical Package for the Social Sciences version 25.0 (SPSS Inc., Chicago, IL, USA). Statistical significance was set at *p* < 0.05. All p-values were two-tailed.

Serum HBsAg and HBV DNA levels were transformed to log_10_ IU/mL for analysis. The time to response was defined as the time between the start of treatment and the first time HBV DNA was undetectable. The data are displayed as mean ± standard deviation or median and interquartile range for numerical data, and as number and percentage for nominal data. Differences between the two groups were assessed using the t-test for numerical parameters, Mann–Whitney test for continuous parameters, and chi-squared test for categorical parameters. A comparison of cumulative rates was performed using the Kaplan–Meier analysis and log-rank test. The factors involved in the VR and HBsAg loss were investigated using Cox’s regression analysis. The hazard ratio (HR) and 95% confidence intervals (CIs) were calculated to evaluate the relative risk confidence. Receiver-operating characteristic curves were generated to examine the diagnostic capability of diverse serum biochemical markers.

## Results

### Baseline characteristics

A total of 128 HBeAg-negative CHB patients were enrolled in this retrospective study. The baseline characteristics of the included patients are shown in Table 1. Eighty-six (67.2%) patients were men. Fifty-four (42.2%) patients had normal ALT. The initial HBV DNA levels of the patients with mildly elevated ALT were significantly higher than those of patients with normal ALT (5.33 vs. 4.66 log_10_ IU/mL; *p* = 0.016). However, age, total bilirubin levels, direct bilirubin levels, white blood cell counts, lymphocyte levels, platelet levels, alpha-fetoprotein levels, and HBsAg levels were similar among patients with normal and mildly elevated ALT. Among CHB patients (n = 54) with normal ALT who underwent FibroScan at baseline, significant fibrosis (F ≥ 2) was found in 44.4% (n = 24) of patients; however, among CHB patients (n = 33) with mildly elevated ALT who underwent FibroScan, 51.5% (n = 17) of patients had significant fibrosis (F ≥ 2). There was no statistical difference in the liver fibrosis stage between groups (*p* = 0.521).

**Table 1:** Baseline characteristics of total patients

Characteristics	Normal ALT (n = 74)	Mildly elevated ALT (n = 54)	*p* value
Age (yr)	41.7 ± 9.2	41.7 ± 9.9	0.989
Male, n, %	44 (59.5%)	42 (77.8%)	0.029
ALT (U/L)	25.39 ± 9.03	67.35 ± 20.56	0.000
AST (U/L)	24.71 ± 8.25	41.94 ± 18.00	0.000
ALB (g/L)	45.25 ± 5.69	44.64 ± 5.39	0.592
TBil (μmol/L)	12.65 ± 5.55	12.53 ± 6.39	0.926
DBil (μmol/L)	4.52 ± 2.15	4.71 ± 2.85	0.700
WBC (× 10^9^/L)	6.31 ± 1.69	6.15 ± 1.25	0.603
LYM (× 10^9^/L)	2.04 ± 0.59	2.12 ± 0.62	0.534
PLT (× 10^9^/L)	214.4 ± 49.38	199.8 ± 56.23	0.197
HGB (g/L)	144.2 ± 17.95	151.06 ± 13.08	0.053
HBV DNA (log_10_IU/mL)	4.66 ± 1.18	5.33 ± 1.74	0.016
HBsAg (log_10_IU/mL)	3.07 ± 0.63	3.14 ± 0.66	0.589
AFP (ng/mL)	3.37 ± 1.87	4.34 ± 3.44	0.147
LSM (kPa)	7.34 ± 2.31	8.22 ± 3.30	0.207

Continuous data are expressed as the mean ± SD

ALT, alanine aminotransferase; AST, aspartate aminotransferase; TBil, total bilirubin; DBil, direct bilirubin; WBC, white blood cell; LYM, lymphocyte; PLT, platelet; HGB, hemoglobin; HBV, hepatitis B virus; HBeAg, hepatitis B e antigen; HBsAg, hepatitis B surface antigen; AFP, alpha-fetoprotein; LSM, liver stiffness measurement

### Virological response

**Fig. 1:**
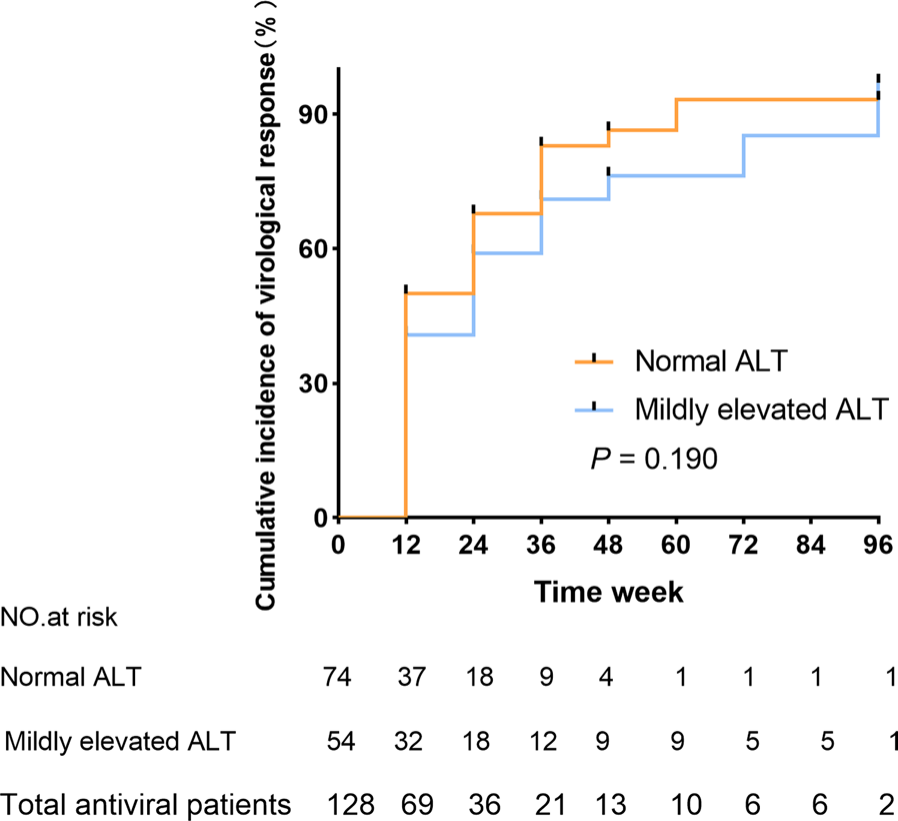
The cumulative incidence of the virological response (VR) of the normal and mildly elevated alanine aminotransferase (ALT) groups. The p-value was determined using log-rank testing

We analysed the outcomes of antiviral therapy for the normal ALT and mildly elevated ALT groups. The total cumulative rates of viral suppression were 46.09%, 64.06%, 77.80%, 81.97%, 89.18%, and 96.39% at weeks 12, 24, 36, 48, 72, and 96, respectively. In the normal ALT group, the cumulative rates of viral suppression were 50.00%, 67.85%, 82.98%, 86.39%, 93.19%, and 93.19% at weeks 12, 24, 36, 48, 72, and 96, respectively. In the mildly elevated ALT group, the cumulative rates of viral suppression were 40.74%, 58.97%, 71.04%, 76.31%, 85.19%, and 97.04% at weeks 12, 24, 36, 48, 72, and 96, respectively. After the log-rank test, no significant difference in the VR was observed between the normal ALT and mildly elevated ALT groups (*p* = 0.190) (Fig. 1).

### Factors associated with the virological response

Univariate and multivariate analyses indicated that the lower HBV DNA levels at week 12 (hazard ratio 0.676; 95% CI 0.560–0.817; *p* < 0.001) and HBsAg levels at week 24 (hazard ratio 0.672; 95% CI 0.485–0.931; *p* < 0.001) were significantly correlated with the VR of all patients at week 96 (Table 2). The relationship between HBV DNA at week 12 and HBsAg levels at week 24 with the VR was evaluated using receiver-operating characteristic curves. The area under the curve for HBV DNA at week 12 for predicting the VR was 0.845, and the optimal HBV DNA cutoff value was 1.56 log_10_ IU/mL. The area under the curve for the HBsAg levels at week 24 was 0.772, and the cutoff value for serum HBsAg was 3.57 log_10_ IU/mL.

**Table 2:** Factors associated with VR* at week 96 in total NAs-treated patients

Variable	Univariate analysis		Multivariate analysis	
	HR (95.0% CI)	*p* value	HR (95.0% CI)	*p* value
Age	1.002 (0.982–1.021)	0.892		
Male (sex)	0.823 (0.549–1.255)	0.366		
Baseline ALT(U/L)	0.994 (0.986–1.002)	0.120		
Baseline AST(U/L)	0.993 (0.983–1.004)	0.209		
HBV DNA (log_10_IU/mL)				
Baseline	0.774 (0.710–0.845)	< 0.001	0.893 (0.780–1.023)	0.104
12 week	0.569 (0.487–0.665)	< 0.001	0.676 (0.560–0.817)	< 0.001
HBsAg (log_10_IU/mL)				
Baseline	0.657 (0.555–0.778)	< 0.001	1.004 (0.688–1.465)	0.985
24 week	0.568 (0.470–0.686)	< 0.001	0.672 (0.485–0.931)	0.017

*Defined as undetectable HBV DNA by sensitive PCR assay during the treatment and follow-up period. NAs, nucleoside/nucleotide analogues; ALT, Alanine aminotransferase; AST, Aspartate aminotransferase; HBV, Hepatitis B virus; HBsAg, hepatitis B surface antigen; HR, Hazard ratio; CI, Confidence interval

### Biochemical response

At weeks 12, 24, 36, 48, 72, and 96, 63.28%, 83.05%, 89.40%, 92.06%, 96.03%, and 96.03% of the total patients showed a biochemical response (ALT < 40 U/L). Of the patients with normal ALT, the cumulative rates of ALT normalization were 72.97%, 88.18%, 92.91%, 95.27%, 95.27%, and 95.27% at weeks 12, 24, 36, 48, 72, and 96, respectively. Of the patients with mildly elevated ALT, the cumulative rates of ALT normalization were 50.00%, 76.09%, 84.78%, 87.83%, 100.00%, and 100.00% at weeks 12, 24, 36, 48, 72, and 96, respectively. There was no significant difference in the ALT normalization rates between groups (*p* = 0.065) (Additional file 1).

### Variations in HBV DNA and HBsAg levels

**Fig. 2:**
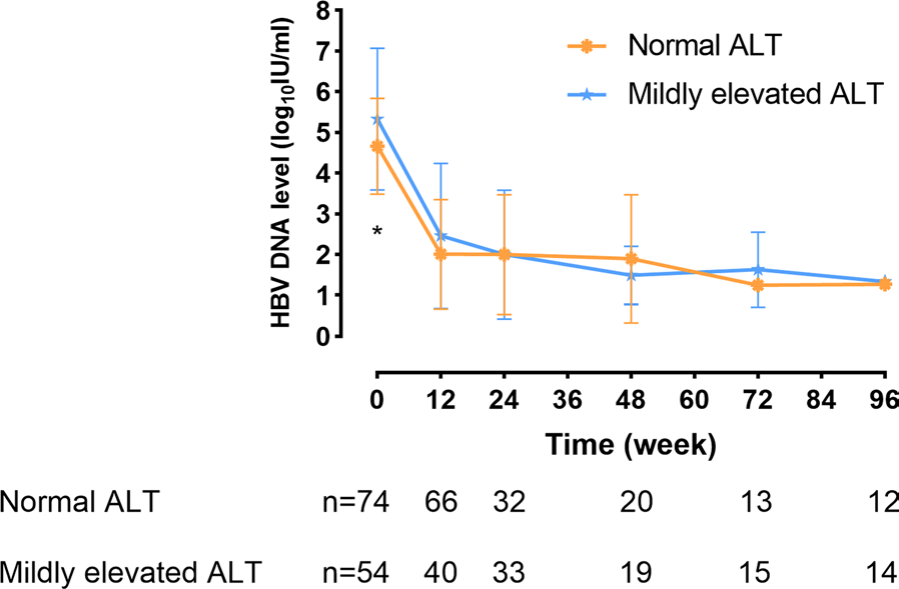
Hepatitis B virus (HBV) DNA levels of patients with normal or mildly elevated alanine aminotransferase (ALT) at each time point (*p < 0.05)

**Fig. 3:**
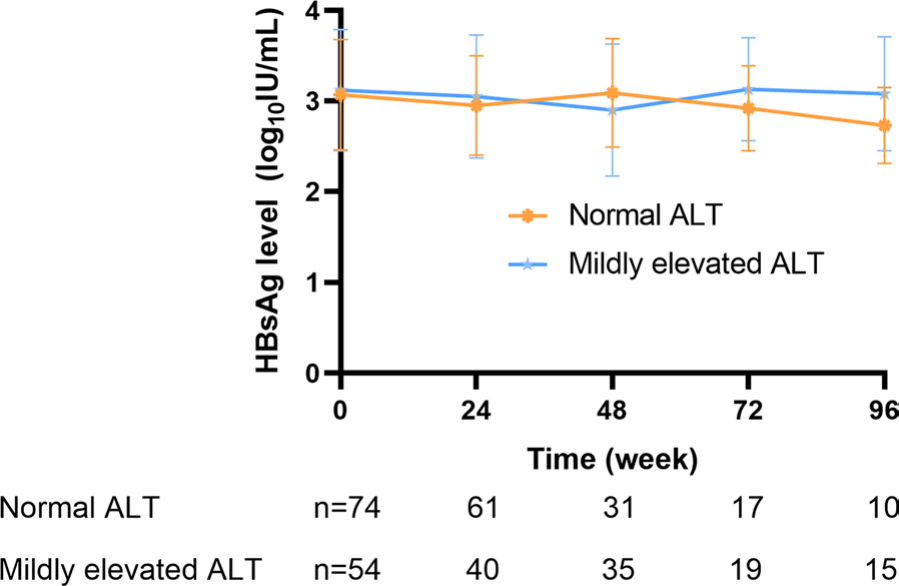
The trend of hepatitis B surface antigen (HBsAg) levels in patients with normal or mildly elevated alanine aminotransferase (ALT) across the study time points

The HBV DNA load significantly decreased among all CHB patients who underwent antiviral treatment during the study period, regardless of ALT levels (Fig. 2). The extent of the decrease in HBV DNA was calculated. For all patients, the mean HBV DNA decreased by 3.28 ± 1.78 IU/mL and 4.08 ± 1.31 IU/mL after 48 weeks and 96 weeks of treatment, respectively. In the normal ALT group, mean HBV DNA decreases of 2.86 ± 1.93 IU/mL and 3.71 ± 1.05 IU/mL were observed after 48 weeks and 96 weeks of treatment, respectively. In the mildly elevated ALT group, these values were 3.73 ± 1.54 and 4.38 ± 1.48 IU/mL, respectively. There was no meaningful effect on the HBV DNA decrease in the two group of patients. The HBsAg loss was not observed during the present study. Furthermore, HBsAg levels did not differ significantly between the normal and mildly elevated ALT groups at any time point. The mean decreases in HBsAg levels at weeks 48 and 96 in these two groups were 0.041 vs 0.259 log_10_ IU/mL (*p* = 0.112) and 0.137 vs 0.222 log_10_ IU/mL (*p* = 0.412), respectively (Fig. 3).

### Improvements in the liver stiffness measurements

**Fig. 4:**
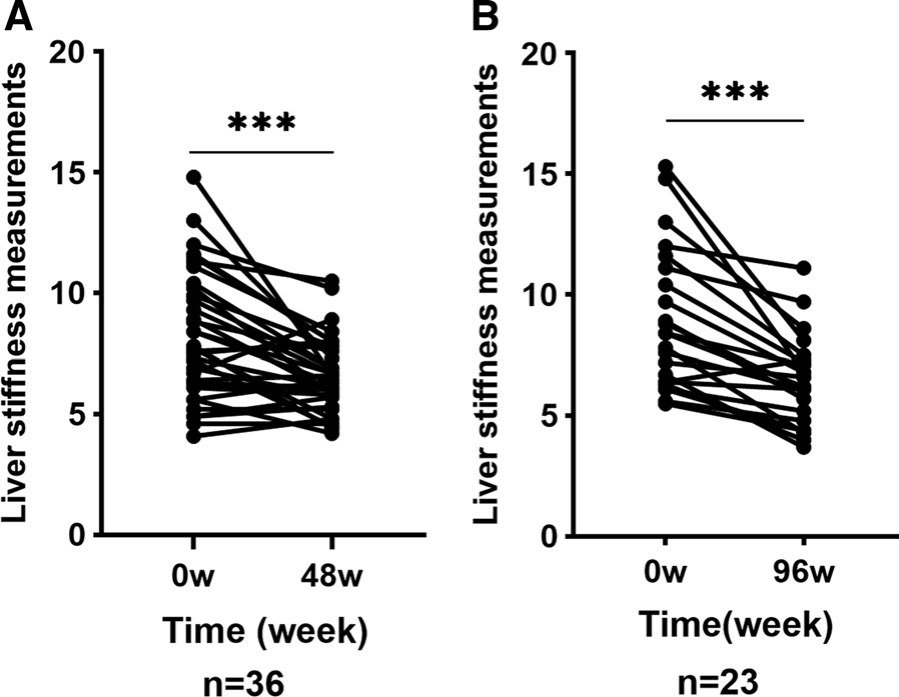
**a** Liver stiffness measurements before treatment and at week 48 **b** Liver stiffness measurements before treatment and at week 96 (***p < 0.001)

There were 36 CHB patients who received antiviral therapy and underwent FibroScan at treatment initiation and week 48. The results showed that the liver stiffness index significantly improved (8.12 kPa vs. 6.57 kPa; *p* < 0.001) (Fig. 4a). FibroScan was also performed for 23 patients at week 96 and showed significant improvement in liver stiffness after treatment (8.87 kPa vs. 6.43 kPa; *p* < 0.001) (Fig. 4b).

## Discussion

This research indicates that NAs treatment can effectively suppress replication of the HBV virus and induced remarkable ALT normalization in HBeAg-negative CHB patients with normal or mildly elevated ALT. The decline in HBV DNA and HBsAg levels between groups was comparable during this study. Liver stiffness significantly improved after antiviral therapy, which may prevent deterioration of liver histology.

The HBV DNA of patients with mildly elevated ALT was higher than that of patients with normal ALT in our study, which was in line with the natural history of chronic HBV infection in patients in the HBeAg-negative chronic HBV infection phase because they have relatively low HBV DNA levels accompanied by persistently normal ALT; however, patients in the HBeAg-negative CHB phase have persistent or fluctuating moderate to high levels of serum HBV DNA as well as fluctuating or persistently elevated ALT [2].

The VR may be important when assessing the antiviral efficacy of NAs for patients. Sustained suppression of HBV replication has been shown to have a protective effect against liver fibrosis progression and related clinical outcomes [28, 29]. The low incidence of the VR to antiviral treatment was one of the reasons for therapy deferment for HBeAg-negative CHB patients with ALT less than the ULN. During our study, however, the VR rate at week 96 in the normal ALT group was parallel to that of the mildly elevated ALT group. During a previous study of HBeAg-negative CHB patients without significantly elevated ALT [16], it was found that patients with normal ALT can achieve a VR similar to that of participants with mildly elevated ALT. Additionally, compared with the reported rates of the VR to NAs of HBeAg-negative CHB patients with ALT more than two-times the ULN (range: 88.0%–96.6% at 24 months) [30–33], our data demonstrated that the VR rates of patients with normal ALT and mildly elevated ALT who received antiviral treatment may be comparable to those of patients with ALT more than two-times the ULN.

Baseline serum HBV DNA levels have been found to correlate with an increased risk of liver necroinflammation and fibrosis in HBeAg-negative CHB patients [34, 35]. During our current study, the HBV viral loads of the two groups decreased notably after antiviral treatment. These observations provide further evidence that HBeAg-negative CHB patients without significantly elevated ALT could benefit from antiviral therapy.

Furthermore, the factors associated with the VR at 96 weeks for HBeAg-negative CHB patients with normal or mildly elevated ALT and the receiver-operating characteristic curve analyses results were evaluated in this study. Our results indicate that when evaluating these patients based on the areas under the receiver-operating characteristic curves for HBV DNA and HBsAg levels during treatment, the HBV viral load at week 12 is a more accurate predictor of the 96-week VR than HBsAg levels at week 24. An HBV viral load < 1.56 log_10_ IU/mL at week 12 indicated favourable virological outcomes. During this study, however, the serum ALT levels failed to predict the VR, thus supporting the conclusion that it is necessary to initiate antiviral therapy for HBeAg-negative CHB patients with normal ALT.

The low rate of HBsAg loss and decreased HBsAg reductions during treatment with NAs remain noteworthy [36, 37]. Previous studies have demonstrated that the cumulative rate of HBsAg clearance for HBeAg-negative CHB patients was 2.2% at 5 years after treatment [38], that the median annual decrease in HBsAg levels over the course of 5 years was 0.098 log IU/ml/year, and that the amplitude of the decrease in the HBsAg level was 5.9% at 2 years after treatment with low genetic barrier NAs [39]. During the current study, no patients achieved loss of HBsAg, and the amplitude of the decrease in the HBsAg level at week 96 was 6.2% for HBeAg-negative CHB patients; these results were similar to those of the aforementioned study.

FibroScan has become a promising and non-invasive method of identifying patients who need antiviral therapy, assessing the effectiveness of antiviral therapy, and predicting the risk of cirrhosis or HCC because of its accuracy and repeatability [27, 40, 41]. However, previous studied have shown that the liver stiffness could be influenced by several factors, such as necroinflammatory activity, ALT elevation, and steatosis [42–44]. These factors might affect liver stiffness through increased portal vein pressure and/or edema of liver tissues associated with hepatocyte necrosis and swelling. Therefore, FibroScan is an important method of assessing liver fibrosis of CHB patients, but it is not a replacement for liver biopsy.

During this study, FibroScan was performed to assess the degree of liver fibrosis. Significant fibrosis was found in 44.4% of CHB patients with normal ALT. The proportion of patients with significant fibrosis in the normal ALT group was equivalent to that of the mildly elevated ALT group, which indicated that the ALT level could not reflect the liver fibrosis stage accurately, and that FibroScan should be routinely used for CHB patients with normal ALT or mildly elevated ALT. In our study, the high efficacy of ALT normalization was observed in two groups after treatment which may further illustrate the reliability of assessing the degree of liver fibrosis with FibroScan after treatment. A favourable finding in our study was the histological amelioration compared with baseline measurements observed for patients after antiviral therapy. Du et al. [45] and Yan et al. [46] reported that fibrosis scores decreased significantly after long-term therapy for HBeAg-negative CHB patients with either normal or elevated ALT. During our study, paired FibroScan measurements exhibited obvious changes in liver stiffness at 48 weeks and 96 weeks. These similar outcomes indicate that antiviral treatment for such patients could result in histological improvements.

### Limitations

This study had some limitations. First, it had a retrospective design. Nevertheless, this study included patients at two teaching hospitals in China, and the sample size was large. Consequently, the data were representative and reliable. Second, a liver biopsy was not been performed for these patients. However, LSMs were used to non-invasively evaluate liver fibrosis. Third, the follow‐up time after antiviral treatment was only 96 weeks. More abundant data regarding the efficacy of antiviral treatment and the loss of HBsAg should be explored by future studies. Despite these limitations, our current findings provide a novel strategy for managing CHB with ALT less than the ULN.

## Conclusions

NAs effectively suppressed HBV DNA in HBeAg-negative CHB patients with normal ALT or mildly elevated ALT and improved the histologic manifestation. Therefore, antiviral therapy is recommended for HBeAg-negative CHB patients with normal ALT. Additionally, significant liver fibrosis is not unusual in HBeAg-negative CHB patients with ALT less than two-times the ULN, and FibroScan should be performed routinely for these patients.

## Supplementary Information

**Additional file 1**. The cumulative incidence of alanine aminotransferase (ALT) normalization in the normal and mildly elevated ALT groups. The p-value was determined using log-rank testing.

## Data Availability

The data supporting the findings of this study are available from the corresponding author upon reasonable request.

## Abbreviations

ALT: Alanine aminotransferase
Anti-HBe: Hepatitis B e-antibody
AST: Aspartate transaminase
CI: Confidence interval
CHB: Chronic hepatitis B
HBeAg: Hepatitis B e-antigen
HBsAg: Hepatitis B surface antigen
HBV: Hepatitis B virus
HCC: Hepatocellular carcinoma
HR: Hazard ratio
LSM: Liver stiffness measurement
NA: Nucleoside/nucleotide analogue
ULN: Upper limit of normal
VR: Virological response
