# Supplementary figures and images for: Effectiveness of antiviral treatment in HBeAg-negative chronic hepatitis B patients with normal or mildly elevated alanine aminotransferase: a retrospective study
Source: BMC Gastroenterol. 2022 Aug 17;22:387. doi: 10.1186/s12876-022-02471-y (PMC9387004; doi:10.1186/s12876-022-02471-y)

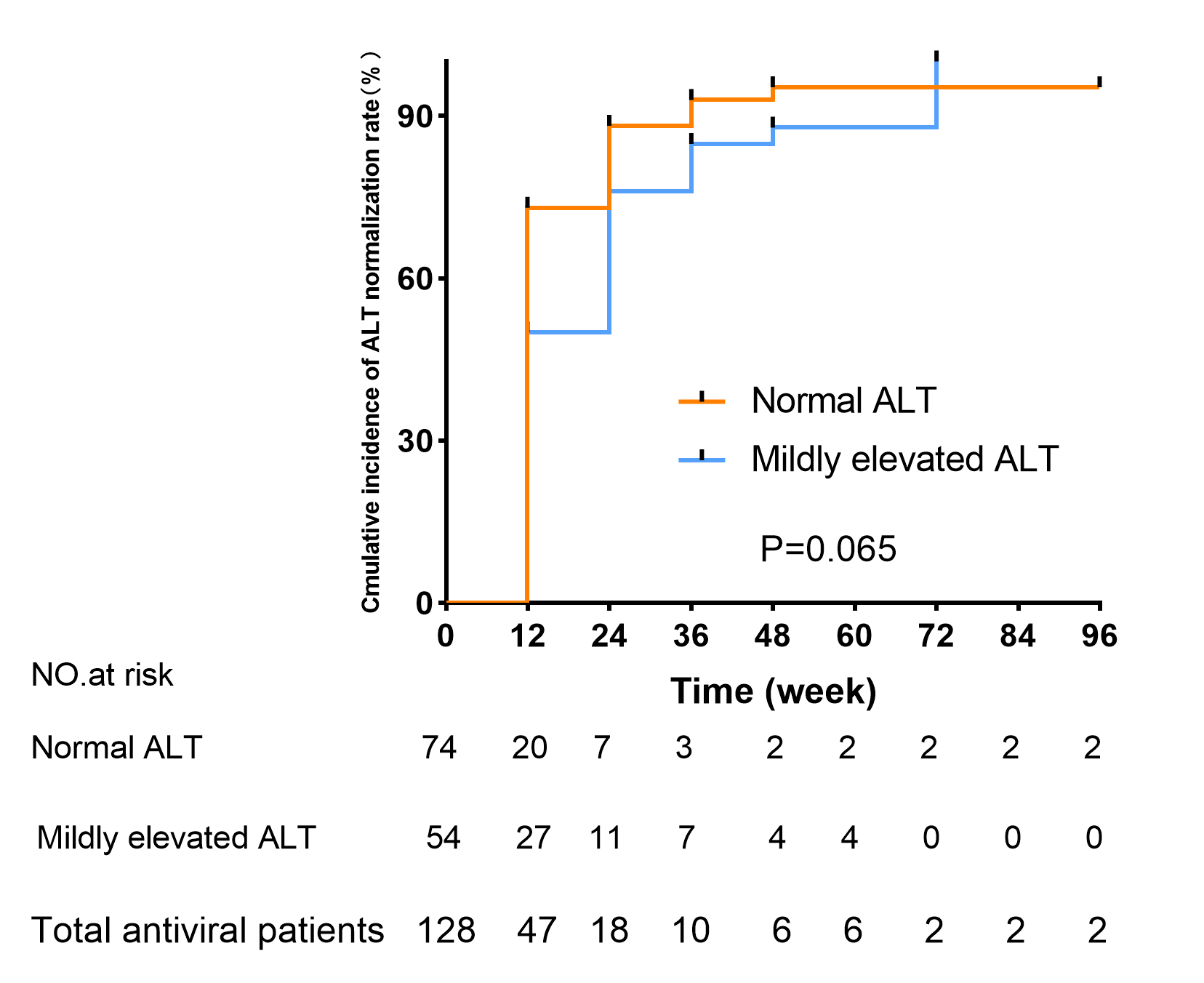

Supplement: Supplementary file 1 — Additional file 1. The cumulative incidence of alanine aminotransferase (ALT) normalization in the normal and mildly elevated ALT groups. The p-value was determined using log-rank testing. [file 12876_2022_2471_MOESM1_ESM.tif]
